# Short-Term Differences in Hospital Resource Utilization and Quality of Care Between Anterior Cervical Discectomy and Fusion and Posterior Cervical Foraminotomy: A National Propensity-Scored Observational Study Utilizing the ACS-NSQIP Database

**DOI:** 10.3390/jcm14186438

**Published:** 2025-09-12

**Authors:** Jaskeerat Gujral, Jonathan H. Sussman, Daniel Gao, Yohannes Ghenbot, John D. Arena, Susanna Howard, Hasan S. Ahmad, John Shin, Jang W. Yoon, Ali K. Ozturk, William C. Welch, Mert Marcel Dagli

**Affiliations:** Department of Neurosurgery, Perelman School of Medicine, University of Pennsylvania, Philadelphia, PA 19104, USAsusanna.howard@pennmedicine.upenn.edu (S.H.);

**Keywords:** discectomy, foraminotomy, length of stay, operative time, radiculopathy, surgical wound infection

## Abstract

**Background/Objective**: Anterior cervical discectomy and fusion (ACDF) or posterior cervical foraminotomy (PCF) are common treatments for cervical radiculopathy. This study compared post-operative outcomes between ACDF and PCF utilizing the American College of Surgeons-National Surgical Quality Improvement Program database. **Methods**: An observational study following STROBE and TRIPOD + AI guidelines compared hospital resource utilization and quality of care between single-level ACDF and PCF (2005–2022). Primary outcomes compared operative time, length of stay (LOS), and post-operative complications. Propensity-scored stabilized inverse probability of treatment weighting adjusted for confounders, specifically demographics, lifestyle-related factors, pre-operative labs, pre-existing comorbidities, and surgery-related factors. Subgroup analysis compared baseline characteristics and outcomes, stratified by 30-day re-admission and re-operation. **Results**: PCF group demonstrated shorter LOS (MD −0.7 days, 95% CI −0.9 to −0.5 days, *p* < 0.001), operative time (MD −32.9 min, 95% CI −35.7 to −30.1 min, *p* < 0.001), higher rate of re-admission associated with overall SSI (PD 1.2%, 95% CI 0.7–1.7%, *p* < 0.001), deep incisional SSI (PD 0.8%, 95% CI 0.4–1.2%, *p* < 0.001), and organ/space SSI (PD 0.3%, 95% CI 0.0–0.5%, *p* = 0.011). Furthermore, the PCF group had greater systemic sepsis (PD 0.8%, 95% CI 0.4–1.3%, *p* < 0.001), overall post-operative SSI (PD 2.8%, 95% CI 2.0–3.6%, *p* < 0.001), superficial SSI (PD 1.9%, 95% CI 1.2–2.5%, *p* < 0.001), and deep incisional SSI (PD 0.8%, 95% CI 0.4–1.2%, *p* < 0.001) rates. Subgroup analysis showed increased early post-operative re-operation rates in the PCF cohort (PD 23.4%, 95% CI 9.5–37.4%, *p* = 0.001) and increased early post-operative re-admission associated with post-operative overall SSI (PD 35.3%, 95% CI 22.7–48.0%, *p* < 0.001). **Conclusions**: Although the PCF cohort demonstrated lower hospital utilization, it had reduced quality of care and increased post-operative complications.

## 1. Introduction

Cervical radiculopathy, defined as the compression of the nerve root in the neck, has been stated to affect 107.3 per 100,000 men and 63.5 per 100,000 women, leading to pain, motor deficits, and sensory deficits [1,2,3].

Anterior cervical discectomy and fusion (ACDF) and posterior cervical foraminotomy (PCF) are two surgical treatment options in the repertoire of both neurological and orthopedic surgeons [4]. Interestingly, there has been a 184% increase in ACDF procedures, while PCF procedures gradually increased by 5.6% between 1992 and 2005 [5]. Both surgical procedures have unique advantages and disadvantages. On one hand, ACDF provides the surgeon with greater access and exposure to intervertebral space and lowers patient discomfort [1]. On the other hand, ACDF might lead to additional risks such as hardware failure, adjacent segment disease, and pseudoarthritis [6]. Although PCF is advantageous in promoting cervical mobility and avoiding some complications associated with an anterior approach and fusion, PCF has been reported to have higher rates of re-operation and post-operative infection [1,4,7,8,9].

While ACDF and PCF are accepted treatment options for cervical radiculopathy, there have been heterogeneous findings in the literature, including operative time, hospital length of stay (LOS), re-admission rates, and re-operation rates [4,10,11,12,13]. These reported discrepancies may be attributed to factors such as underpowered studies, limited generalizability, and differing follow-up time points. Interestingly, there is no literature on the breakdown of re-admission associated with early post-operative 30-day overall SSI and sub-categories for ACDF and PCF, most likely due to the required sample sizes for causal inference. Addressing these conflicting findings and gaps with a strongly powered study design appropriately adjusted for imbalances between groups could provide more robust insight into the quality of care and hospital resource utilization between ACDF and PCF.

Thus, we used the American College of Surgeons-National Surgical Quality Improvement Program (ACS-NSQIP) database to investigate differences in hospital resource utilization and quality of care between ACDF and PCF. Specifically, we focused on early re-admissions related to 30-day post-operative surgical site infections (SSIs) and their sub-categories, overall and broken down by severity. To account for imbalances in baseline characteristics between the groups, we applied propensity score stabilized inverse probability weighting.

## 2. Materials and Methods

### 2.1. Guidelines

The design and reporting of this study adhered to the Strengthening the Reporting of Observational Studies in Epidemiology (STROBE) and Transparent Reporting of a multivariable prediction model for Individual Prognosis Or Diagnosis + Artificial Intelligence (TRIPOD + AI) guidelines [14,15].

### 2.2. Data Source and Patient Selection

This retrospective study screened the ACS-NSQIP database. Database records of surgeries conducted from 2005 to 2022 were reviewed, and included adult patients (age ≥ 18 years old) who underwent single-level ACDF or PCF. The ACS-NSQIP database consists of a voluntary consortium of participating hospitals. Specifically, the database includes large academic hospitals, community hospitals, and urban hospitals, while excluding non-federal psychiatric hospitals, non-federal long-term care hospitals, and hospital units of institutions [16]. The cases were filtered utilizing Current Procedural Terminology (CPT) codes of primary procedure, either ACDF or PCF. Specifically, CPT code 63020 was used for PCF, and 22551 was used for ACDF. Patients and cases were excluded if they met any of the following criteria: under 18 years of age, underwent multi-level surgery, missing data of extracted variables in their respective years of collection, involved additional procedures, or were performed by physicians other than neurosurgeons or orthopedic surgeons.

### 2.3. Outcomes

The primary outcome of this study was the investigation of differences in hospital resource utilization, which was defined as operative time and hospital LOS based on the prior literature [17,18]. Quality of care included overall 30-day re-admission, re-admission associated with early post-operative 30-day overall SSI and sub-categories, and 30-day re-operation rates, between single-level ACDF and PCF. Subgroup analysis was performed for overall 30-day re-admission and re-operation rates. Secondary outcomes included an exploration of post-operative complications and outcomes.

### 2.4. Variables

The following variables were extracted for each eligible patient: demographics (age, sex, race, Hispanic ethnicity), lifestyle-related factors (body-mass-index [kilograms per square meter {kg/m^2^}], smoking status, diabetes), pre-operative labs (albumin [grams per deciliter {g/dL}], white blood cells (WBCs) (1000/mL), hematocrit (HCT) (%), platelets [1000 cells per microliter {1000/mL}], hemoglobin [grams per deciliter] {g/dL}, partial thromboplastin time (PTT) [seconds {s}]), INR, blood urea nitrogen (BUN) [milligrams per deciliter] {mg/dL}, creatinine [milligrams per deciliter {mg/dL}], albumin (g/dL), pre-existing comorbidities (congestive heart failure (CHF), disseminated cancer, steroid use, bleeding disorder, chronic obstructive pulmonary disease (COPD), pulmonary embolism (PE), dialysis, weight loss, renal failure, dyspnea, cerebrovascular accident, myocardial infarct), and surgery-related factors (surgery-related factors) (surgery specialty, operative time (minutes), estimated blood loss (EBL) total [milliliter]), transfer from home status, functional status, American Society of Anesthesiologists’ (ASA) score, transfusion, post-operative complications (thromboembolic, cardiovascular, renal, pulmonary, neurologic, surgical site), central nervous system cerebrovascular accident (stroke), occurrence hospital length of stay (LOS) (days), post-operative complications (pulmonary embolism, failure to wean from ventilator (>48 h), cardiac arrest requiring cardiopulmonary resuscitation, myocardial infarction, deep vein thrombosis (DVT) or thrombophlebitis, systemic sepsis, superficial incisional SSI, deep incisional SSI, organ/space SSI), overall 30-day re-admission, re-admission associated with early post-operative 30-day overall SSI and sub-categories (superficial incisional SSI, deep incisional SSI, organ/space SSI), unplanned return to the operating room for any reason within 30 days, and re-operation. Overall SSI rates for post-operative complications and re-admission associated with early post-operative 30-day overall SSI and sub-categories related to suspected reasons were calculated by pooling rates of superficial incisional, deep incisional, and organ/space SSI. Hospital resource utilization, crucial for effectively managing resources to ensure proper workflow and resource allocation, can be determined by factors including LOS and operative time [19,20]. Hospital resource was defined by LOS and operative time. Quality of care was measured by overall re-admission rates and re-operation rates as proxies, according to the prior literature [21,22,23,24,25,26,27].

### 2.5. Missing Data

Prevalence of missing data constituted under 4% of the final dataset. Missing values were imputed using a multiple imputation approach, leveraging a random forest machine learning algorithm. Each imputation iteration utilized a different random state to account for uncertainty.

### 2.6. Statistics

Baseline characteristics were summarized with descriptive statistics. Propensity scores (PS) for ACDF and PCF were calculated using logistic regression and included the following covariates: demographics (age, sex, race, Hispanic ethnicity), lifestyle-related factors (BMI, smoking status, diabetes), pre-operative labs (WBC (1000/mL)), HCT (%), platelets (1000/mL), PTT (s), INR, BUN (mg/dL), creatine (mg/dL), albumin (g/dL), pre-existing comorbidities (CHF, disseminated cancer, steroid use, bleeding disorder, COPD, dialysis, weight loss, renal failure, dyspnea), and surgery-related factors (operative time, transfer from home status, functional status, ASA score, transfusion, specialty). To mitigate confounding variables, stabilized inverse probability treatment weighting (SIPTW) was applied, which allowed for the estimation of the average treatment effect across the entire patient cohort and accounted for the marginal distribution of observed covariates. A standardized mean difference (SMD) of less than 0.10 denoted a good balance between the two groups. Unadjusted categorical outcomes were analyzed using the Pearson χ^2^ test or when at least 1 of the cells of the contingency table had an expected *n* < 5, Fisher’s exact probability test, continuous non-normal data using the Mann–Whitney U test, and continuous normal data using the unpaired *t*-test. Stabilized weighted regression models were used to calculate *p* values for weighted analysis. The treatment effect was quantified as weighted mean difference (MD) for continuous and difference in proportions (PD) for categorical data. Additionally, 95% confidence intervals (CIs) were calculated using weighted means, variances, and standard errors. *p* values for primary outcomes were adjusted for multiplicity within each section using the Benjamini–Hochberg correction to control the false discovery rate (FDR) at a level of 0.05. Widths of all CIs, subgroup, and exploratory analysis of secondary outcomes were not adjusted for multiplicity. Subgroup analysis was performed, stratifying by 30-day re-admission and 30-day re-operation. All statistical analysis and the figures were conducted and generated using Python, version 3.9.6 (Python Foundation, Wilmington, DE, USA).

## 3. Results

### 3.1. Study Population

**Figure 1:**
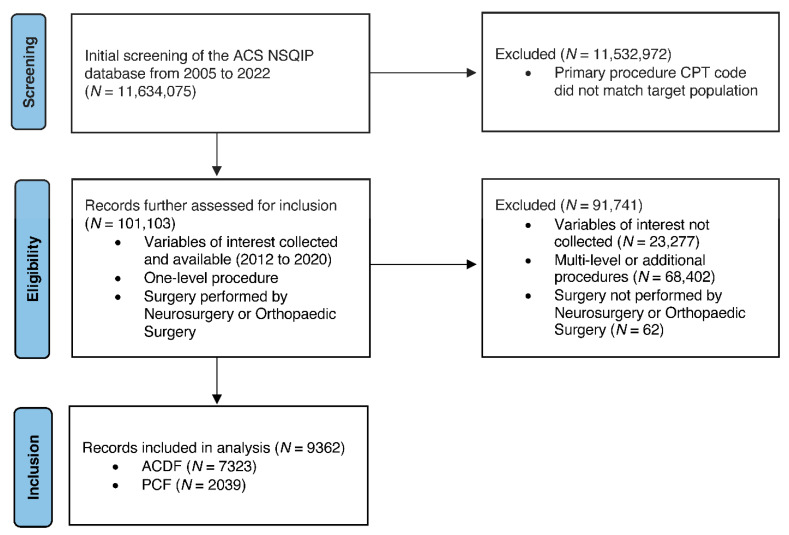
STROBE Checklist. Flowchart detailing criteria and process of patient selection from ACS-NSQIP database.

**Figure 2:**
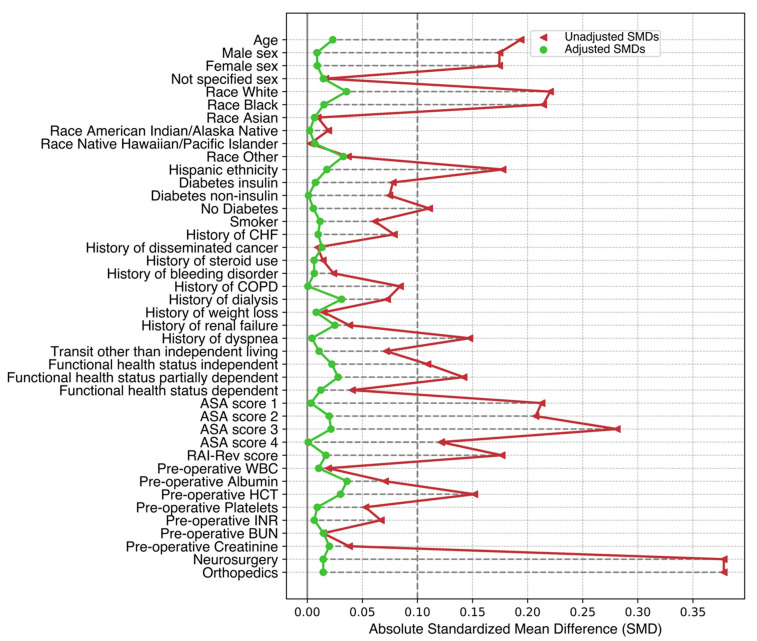
A love plot of the unadjusted and adjusted SMD for anterior cervical discectomy and fusion vs. posterior cervical foraminotomy. This love plot exhibits adjusted and unadjusted standardized mean differences (SMDs) for baseline characteristics between anterior cervical discectomy and fusion (ACDF) and posterior cervical foraminotomy (PCF). This plot highlights the balance before and after adjustment; the threshold is set to an absolute SMD < 0.1, representing an acceptable balance.

Following initial screening of the NSQIP database (N = 11,634,075), our study cohort included 9424 patients (ACDF: 7323; PCF: 2039) who underwent single-level ACDF or PCF (Figure 1; Table 1). Prior to adjustment, 19 of 45 characteristics had an absolute SMD (ASMD) greater than 0.1. Post-adjustment with SIPTW, both groups were well balanced on all baseline characteristics (Figure 2). The effective sample size in our SIPTW analysis was 7331 in the ACDF and 2049 in the PCF group.

### 3.2. Primary Outcomes

After adjusting for multiplicity, statistically significant differences were observed, with the PCF group demonstrating a shorter hospital LOS (MD −0.7 days with 95% CI −0.9 to −0.5 days, *p* < 0.001) and operative time (MD −32.9 min with 95% CI −35.7 to −30.1 min, *p* < 0.001). Additionally, we examined re-admission associated with early post-operative 30-day overall SSI and sub-categories. The PCF group demonstrated a higher rate of re-admission associated with post-operative overall SSI (PD 1.2% with 95% CI 0.7 to 1.7%, *p* < 0.001), superficial SSI (PD 0.1% with 95% CI −0.1 to 0.3%, *p* = 0.135), deep incisional SSI (PD 0.8%, with 95% CI 0.4 to 1.2%, *p* < 0.001), and organ/space SSI (PD 0.3% with 95% CI 0.0% to 0.5%, *p* = 0.011) than the ACDF group (Table 2).

### 3.3. Secondary Outcomes

The PCF group experienced greater systemic sepsis occurrences (PD 0.8% with 95% CI 0.4 to 1.3%, *p* < 0.001), overall post-operative SSI (PD 2.8% with a 95 CI to 2.0 to 3.6%, *p* < 0.001), post-operative superficial SSI (PD 1.9% with a 95% CI 1.2 to 2.5%, *p* < 0.001), and deep incisional SSI (PD 0.8% with a 95% CI 0.4 to 1.2%, *p* < 0.001). There were no statistically significant differences in any of the other post-operative complications analyzed in this study (Table 3).

### 3.4. Subgroup Analysis

We investigated differences in baseline characteristics between ACDF and PCF cohorts stratified by 30-day re-admission and 30-day re-operation. We found a statistically significant difference with the PCF group proportion of White and Black patients experiencing 30-day re-admission, (PD −19.6% with a 95% CI −32.9 to −6.4%, *p* = 0.001) and (PD 12.0% with a 95% CI −0.6 to 23.4%, *p* = 0.032), respectively. Furthermore, the PCF group demonstrated a greater BMI for patients who had 30-day re-admission than the ACDF group (PD 2.1% with a 95% CI 0.1 to 4.0%, *p* = 0.042). We also found a greater number of smokers in the ACDF group compared to the PCF group who underwent 30-day re-admission (PD 14.8% with a 95% CI 1.0 to 28.5%, *p* = 0.034). For 30-day re-operation, we observed that the ACDF cohort was older (MD −6.2 years with a 95% CI −10.8 to −1.6 years, *p* = 0.003) with a greater proportion of females (PD 18.0% with a 95% CI 0.2 to 35.8%, *p* = 0.046) and males (PD −18.0% with a 95% CI −35.8 to −0.2%, *p* = 0.046), respectively. We also observed that the ACDF cohort had a greater proportion of White and Black patients undergoing 30-day re-operation than PCF, (PD −20.7% with a 95% CI −38.3 to −3.1%, *p* = 0.018) and (PD 23.3% with a 95% CI 6.7 to 39.8%, *p* = 0.003), respectively. Interestingly, the ACDF group demonstrated a greater proportion of insulin-dependent diabetes (PD 12.5% with a 95% CI −1.1 to 26.1%, *p* = 0.032), no diabetes (PD −19.3% with a 95% CI −36.1 to −2.5%, *p* = 0.018), and RAI-rev score (MD −3.0 points with a 95% CI −5.5 to −0.6 points, *p* = 0.009). The PCF group had a statistically significant difference in BMI (MD 3.5 kg/m^2^ with 95% CI 1.0 to 5.9 kg/m^2^, *p* = 0.01) (Table 4).

We also determined differences in outcomes between ACDF and PCF cohorts stratified by 30-day re-admission and 30-day re-operation. For 30-day re-admission, statistically significant differences in the ACDF were found in longer operative time (MD −52.7 min with a 95% CI −68.7 to −36.8 min, *p* < 0.001) and greater proportion of re-operation (PD 23.4% with a 95% CI 9.5 to 37.4%, *p* = 0.001). The PCF group demonstrated a greater sepsis rate (PD 24.3% with a 95% CI 13.3 to 35.3%, *p* < 0.001), overall 30-day SSI (PD 33.5% with a 95% CI 20.5 to 46.4%, *p* < 0.001), deep incisional SSI (PD 24.7% with a 95% CI 13.4 to 36.0%, *p* < 0.001), 30-day re-admission associated with overall SSI (PD 35.3% with a 95% CI 22.7 to 48.0%, *p* < 0.001), 30-day re-admission associated with deep incisional SSI (PD 23.1% with a 95% CI 12.0 to 34.3%, *p* < 0.001), 30-day re-admission associated with open/space SSI (PD 7.7% with a 95% CI 0.3 to 15.0%, *p* = 0.014), and 30-day re-operation (PD 23.4% with a 95% CI 9.5 to 37.4%, *p* = 0.001). For 30-day re-operation, we observed that the ACDF group had longer operative time (MD −64.2 with 95% CI −87.3 to −41.1 min, *p* < 0.001) and a greater proportion of any 30-day re-admission (MD 45.6% with 95% CI 32.0 to 59.2%, *p* = 0.001) than that of the PCF group. Interestingly, the PCF group had a longer LOS (MD −5.4 with 95% CI −7.8 to −3.0 days, *p* = 0.001), systemic sepsis (PD 36.2% with 95% CI 20.3 to 52.2%, *p* < 0.001), any overall SSI (PD 52.1% with a 95% CI 36.0 to 68.2%, *p* < 0.001), superficial SSI (PD 9.6% with 95% CI −1.3 to 20.6%, *p* = 0.029), deep incisional SSI (PD 33.7% with 95% CI 17.9 to 49.4%, *p* < 0.001), organ/space SSI (PD 13.1% with a 95% CI 0.5 to 25.8%, *p* = 0.016), 30-day re-admission associated with overall SSI (PD 51.6% with a 95% CI 35.6 to 67.7%, *p* < 0.001), and 30-day re-admission associated with deep incisional SSI (PD 31.9% with 95% CI 16.4 to 47.4%, *p* < 0.001) (Table 5).

## 4. Discussion

To the best of our knowledge, this was the largest propensity-scored stabilized inverse probability-weighted observational study, utilizing the ACS-NSQIP to primarily assess the differences in hospital resource utilization and quality of care after ACDF and PCF procedures. It was the first to investigate differences in early post-operative re-admission rates associated with post-operative SSI, overall and broken down by severity, between both procedures. Secondary outcomes included an exploration of post-operative complications. Subgroup analysis evaluated differences in baseline characteristics and outcomes, stratified by 30-day re-admission and 30-day re-operation.

There have been heterogeneous findings in the literature, including but not limited to, operative time, hospital length of stay (LOS), early post-operative re-admission rates associated with post-operative overall SSI and its sub-categories, and re-operation rates [4,10,11,12,13]. Santangelo et al. found no significant differences in operation time between the two procedures, whereas Fang et al. reported that the PCF group was associated with a shorter operative time compared to the ACDF group [10,11]. For LOS, one study determined that LOS was comparable between the ACDF and PCF groups [4], while another reported that the ACDF group experienced a greater LOS than the PCF group [10]. On one hand, Santangelo et al. showed a greater re-admission rate at 90-day and 1-year time points in the ACDF group, compared to the PCF group [10]. On the other hand, Ng et al. reported a greater early post-operative 30-day re-admission rate in the PCF group compared to the ACDF group in their unadjusted data, which was insignificant in their adjusted data [9].

While some studies reported no significant differences in the re-operation rate 2 years post-operatively [16,23], other studies demonstrated a lower re-operation rate in the ACDF group compared to the PCF group [11,13]. At the time of synthesis, there was no literature on the differences in the early post-operative re-admission rate overall SSI and sub-categories for ACDF and PCF.

In our study, we observed that the PCF group had a shorter operative time and hospital LOS than the ACDF group, translating to lower resource utilization. This may reflect the increased complexity of the anterior approach [28,29]. These results highlight the potential of PCF to minimize hospital resource utilization, inform clinicians and key stakeholders, allow for the optimization of resource allocation, and maintain good workflow.

Additionally, we demonstrated that the overall 30-day re-admission rate was comparable between the ACDF and PCF groups. However, the PCF cohort had a greater 30-day re-admission rate associated with post-operative overall SSI, post-operative deep incisional SSI, and post-operative organ/space SSI than the ACDF cohort. These findings translate to lower quality of care in the PCF cohort. This may be explained by the nature of the procedure, where anterior fusions involve less extensive muscle dissection for bone exposure [30]. Another explanation for this result may be due to sweat contamination that is induced by Staphylococcus epidermidis, preventing wound healing and resulting in SSI [31]. Interestingly, the ACDF and PCF groups were comparable in the overall 30-day re-operation rates. These findings underscore the nuanced differences in quality of care metrics between procedures, offering valuable insights for key stakeholders of expectations and guiding resource allocation.

We also noted increased post-operative complications, particularly sepsis, to be more prevalent in the PCF group than in the ACDF group. Additionally, the PCF group experienced greater post-operative overall SSI, superficial SSI, and deep incisional SSI. This may be attributed to a higher likelihood of developing infection due to the posterior approach and potential factors, such as sweat contamination, preventing wound healing and resulting in SSI [31,32].

Subgroup analysis stratified by 30-day re-admission revealed differences in the proportion of baseline characteristics with a decreased proportion of White patients, an increased proportion of Black patients, decreased BMI, and decreased rate of smoking in the ACDF group, compared to the PCF group. This may be due to racial disparities and differences in lifestyle-related factors, or residual confounding factors, which can be considered hypothesis-generating. Stratified by early post-operative re-operation, the ACDF group showed a statistically significant increase in age, male gender, white race, decrease in Black race, decrease in insulin-dependent diabetes, decrease in BMI, decrease in partially dependent functional status, and increase in RAI-rev score compared to the PCF group. These findings suggest that there may be residual confounding variables, which should also be considered hypothesis-generating.

Subgroup analysis of outcomes stratified by 30-day re-admission revealed that the ACDF group had longer operative times but lower rates of post-operative systemic sepsis, post-operative deep incisional SSI, and early post-operative re-admission associated with post-operative overall SSI than the PCF group. Interestingly, the ACDF cohort had higher rates of early post-operative re-admission associated with post-operative deep incisional SSI, early post-operative re-admission associated with post-operative organ/space SSI, and early post-operative re-operation rates compared to the PCF group. These findings mainly aligned with the main analysis, except for early post-operative re-admission associated with post-operative overall SSI and early post-operative re-operation rates. This finding implies that re-admitted patients for post-operative overall SSI and early post-operative re-operation have a higher risk of complications and may require monitoring. Stratified by 30-day re-operation, the ACDF group had longer operative time, LOS, and decreased post-operative systemic sepsis, post-operative SSI rates, early post-operative re-admission associated with post-operative overall SSI, and early post-operative re-admission associated with post-operative deep incisional SSI. These findings were consistent with the main analysis. Although primary analysis revealed no statistically significant differences in re-operation, subgroup analysis stratified by 30-day re-admission demonstrated statistically significant differences.

Our study compared patient outcomes between anterior vs. posterior approaches, rather than the technique itself. The observed greater SSI infection rate in the PCF cohort may be attributed to wound healing complications. However, there is a subgroup of patients who would benefit more from PCF than from ACDF. Although our study demonstrates the short-term trade-offs between the two approaches, the study does not recommend against PCF in selected patients.

This study contributes to the existing literature by delineating complications that may impact certain quality of care and hospital utilization metrics. Although we described re-admission and re-operation as quality of care, with operative time and LOS as hospital resource utilization, these variables are all clinically relevant patient outcomes that influence hospital system benchmarking for resource utilization. For instance, while PCF may have shorter operative time and length of stay, there may be an increased risk of post-operative infections. The granularity presented may inform clinicians of these trade-offs, guiding more accurate decision making.

## 5. Limitations

Even though the present study benefited by extensive balancing of baseline characteristics, this study must be interpreted within the context of its limitations. Firstly, although we adjusted for confounding variables in our study with SIPTW, limitations inherent in its retrospective design remain. Secondly, our study was limited to early post-operative follow-up due to the ACS-NSQIP not collecting beyond this time point. Due to this limitation, it was not possible to assess long-term outcomes, such as functional disorders or paresthesia. Accuracy limitations and limitations regarding the granularity of the data can exist with large administrative national databases. Specifically, the NSQIP database does not collect granular details on surgical equipment, medical products, and surgeon experience. These factors were not accounted for and may influence variables, such as SSI. Thirdly, both surgeon and patient preferences may not have been sufficiently controlled for, due to these variables not being available in the ACS-NSQIP database. Fourthly, our subgroup analysis employed a within-subset approach using weights from the main analysis, which might have introduced residual confounding. Fifthly, the ACS-NSQIP database reports inpatient and outpatient procedures that are performed only in hospitals participating in providing data to the NSQIP organization, instead of just ambulatory surgical settings. Due to this, our findings are generalizable to hospital centers rather than just ambulatory surgical settings as the differences in peri-operative protocols may not be accounted for. Additionally, the NSQIP database does not capture data on prior cervical spine surgeries and patient reported outcomes, which may pose as a risk of bias and confounding factors. Even though statistically sound, a dedicated analysis, for instance, multivariate models, investigating 30-day re-admission and 30-day re-operation might offer additional insight into the role of predictors. Lastly, our study primarily focused on patients who underwent single-level ACDF and PCF procedures, which limits the generalization of our findings.

## 6. Conclusions

We demonstrated that the PCF group had a shorter operative time and LOS, but a higher rate of 30-day re-admission associated with post-operative overall SSI, deep incisional SSI, and organ/space SSI than the ACDF group. Subgroup analysis stratified by 30-day re-operation showed an increased LOS for the ACDF cohort than the PCF cohort. Additionally, subgroup analysis stratified by 30-day re-admission demonstrated an increased early post-operative re-operation rate compared to the PCF group. Future studies should investigate long-term differences, with comparison between control and multi-level surgical procedures, comparison in outcomes between hospital vs. ambulatory surgical settings, socioeconomic data, and the role of predictors for early post-operative re-admission and re-operation.

## Figures and Tables

**Table 1:** Baseline characteristics of patients receiving ACDF and PCF.

Characteristics	Before Weighting			After Weighting		
	ACDF (*N* = 7323)	PCF (*N* = 2039)	ASMD	ACDF (*N* = 7332)	PCF (*N* = 2038)	ASMD
Age (years)	55.2 ± 12.1	52.9 ± 12.3	0.194	54.7 ± 12.2	55.0 ± 12.5	0.023
Female sex	3528 (69.6)	806 (39.5)	0.175	3395 (46.3)	953 (46.8)	0.009
Male sex	3794 (51.8)	1233 (60.5)	0.175	3936 (53.7)	1085 (53.2)	0.009
Not specified	1 (0.0)	-	0.017	1 (0.0)	-	0.015
Race						
White	5430 (74.1)	1696 (83.2)	0.222	5577 (76.1)	1519 (74.5)	0.036
Black	849 (11.6)	114 (5.6)	0.215	755 (10.3)	219 (10.7)	0.015
Asian	148 (2.0)	44 (2.2)	0.010	149 (2.0)	40 (2.0)	0.007
American Indian/Alaska Native	33 (0.5)	12 (0.6)	0.019	35 (0.5)	10 (0.5)	0.002
Native Hawaiian/Pacific Islander	30 (0.4)	8 (0.4)	0.003	30 (0.4)	9 (0.4)	0.007
Other	5 (0.1)	-	0.037	4 (0.1)	-	0.033
Hispanic ethnicity	468 (6.4)	55 (2.7)	0.178	409 (5.6)	105 (5.2)	0.018
Diabetes status						
Diabetes insulin	459 (6.3)	92 (4.5)	0.078	431 (5.9)	116 (5.7)	0.007
Diabetes non-insulin	806 (11.0)	179 (8.8)	0.075	772 (10.5)	214 (10.5)	0.001
No diabetes	6058 (82.7)	1768 (86.7)	0.111	6129 (83.6)	1708 (83.8)	0.006
BMI (kg/m^2^)	30.6 ± 6.9	29.8 ± 6.1	0.112	30.4 ± 6.8	30.4 ± 6.4	0.003
Smoker	1877 (25.6)	469 (23.0)	0.061	1835 (25.0)	500 (24.5)	0.012
Prior medical history						
CHF	32 (0.4)	1 (0.0)	0.079	26 (0.4)	6 (0.3)	0.010
Disseminated cancer	18 (0.2)	6 (0.3)	0.009	18 (0.2)	4 (0.2)	0.013
Steroid use	211 (2.9)	54 (2.6)	0.014	206 (2.8)	59 (2.9)	0.006
Bleeding disorder	86 (1.2)	19 (0.9)	0.024	82 (1.1)	21 (1.0)	0.006
COPD	337 (4.6)	61 (3.0)	0.084	313 (4.3)	87 (4.3)	0.001
Dialysis	36 (0.5)	2 (0.1)	0.073	30 (0.4)	5 (0.2)	0.031
Weight loss	16 (0.2)	6 (0.3)	0.015	17 (0.2)	4 (0.2)	0.008
Renal failure	13 (0.2)	1 (0.0)	0.038	11 (0.2)	1 (0.0)	0.025
Dyspnea	346 (4.7)	42 (2.1)	0.148	304 (4.1)	86 (4.2)	0.004
Transfer from non-home	176 (2.4)	29 (1.4)	0.072	161 (2.2)	48 (2.4)	0.011
Functional status						
Independent	7106 (97.0)	2011 (98.6)	0.109	7140 (97.4)	1977 (97.0)	0.022
Partially dependent	160 (2.2)	11 (0.5)	0.142	134 (1.8)	45 (2.2)	0.028
Dependent	20 (0.3)	2 (0.1)	0.041	17 (0.2)	6 (0.3)	0.012
ASA class						
1	250 (3.4)	172 (8.4)	0.214	330 (4.5)	90 (4.4)	0.03
2	3626 (49.5)	1220 (59.8)	0.208	3791 (51.7)	1034 (50.7)	0.020
3	3233 (44.1)	623 (30.6)	0.284	3024 (41.2)	862 (42.3)	0.022
4	201 (2.7)	22 (1.1)	0.122	175 (2.4)	48 (2.4)	0.001
RAI-rev score	17.3 (5.7)	16.3 (5.5)	0.177	17.1 (5.7)	17.2 (5.9)	0.017
Pre-operative lab values						
WBC (1000/mL)	7.6 ± 2.5	7.6 ±4.3	0.019	7.6 ± 2.5	7.6 ± 3.3	0.010
HCT (%)	41.5 ± 4.2	42.2 ± 4.1	0.152	41.7 ± 4.2	41.5 ± 4.7	0.030
Platelets (1000/mL)	250.3 ± 65.9	246.9 ± 61.1	0.053	249.6 ± 64.9	250.2 ± 64.3	0.009
PTT (s)	29.2 ± 3.6	29.0 ± 3.1	0.061	29.2 ± 3.6	29.2 ± 3.3	0.006
INR	1.0 ± 0.2	1.0 ± 0.1	0.067	1.0 ± 0.1	1.0 ± 0.1	0.006
BUN (mg/dL)	15.9 ± 7.1	15.8 ± 5.5	0.014	15.9 ± 7.2	16.0 ± 6.1	0.015
Creatinine (mg/dL)	0.9 ± 0.6	0.9 ± 0.3	0.038	0.9 ± 0.5	0.9 ± 0.4	0.020
Albumin (g/dL)	4.1 ± 0.3	4.1 ± 0.3	0.071	4.1 ± 0.3	4.1 ± 0.3	0.036
Specialty						
Neurosurgery	5303 (72.4)	1785 (87.5)	0.385	5551 (75.7)	1556 (76.3)	0.015
Orthopedic surgery	2020 (27.6)	254 (12.5)	0.385	1781 (24.3)	483 (23.7)	0.015

Data are mean ± standard deviation or number of patients (%). ASA: American society of anesthesiologists’ physical status; ASMD: absolute standardized mean difference; BMI: body-mass-index; BUN: blood urea nitrogen; CHF: congestive heart failure; COPD: chronic obstructive pulmonary disease; HCT: hematocrit; INR: international normalized ratio; PTT: partial thromboplastin time; RAI-rev score: revised risk analysis index score; WBC: white blood cells.

**Table 2:** Primary outcomes in ACDF and PCF patients.

Variable	ACDF (*N* = 7332)	PCF (*N* = 2038)	Difference Unadjusted (95% CI)	*p* Value ^a^
Operative time (min)	129.5 ± 70.7	96.6 ± 53.0	−32.9 (−35.7 to −30.1)	<0.001 *
Length of stay (days)	2.3 ± 6.0	1.6 ± 3.2	−0.7 (−0.9 to −0.5)	<0.001 *
Any re-admission	175 (2.4)	64 (3.1)	0.8% (−0.1% to 1.6%)	0.094
Re-admission due to SSI	12 (0.2)	27 (1.3)	1.2% (0.7% to 1.7%)	<0.001 *
Superficial incisional	3 (0.0)	3 (0.1)	0.1% (−0.1% to 0.3%)	0.135
Deep incisional	6 (0.1)	17 (0.8)	0.8% (0.4% to 1.2%)	<0.001 *
Organ/space	3 (0.0)	6 (0.3)	0.3% (0.0% to 0.5%)	0.011 *
Re-operation	125 (1.7)	39 (1.9)	0.2% (−0.5% to 0.9%)	0.660

Data is from the weighted analysis. Data are mean ± standard deviation or number of patients (%). The differences are mean difference and difference in proportions, along with their 95% confidence intervals. CI: confidence intervals; SSI: surgical site infection. ^a^ Adjusted *p* values: Benjamini–Hochberg correction with a false discovery rate (FDR) of 0.05 was applied to adjust for multiplicity. The widths of the confidence intervals were not adjusted for multiple testing and may not be used in place of hypothesis testing. * Significant *p* values after adjusting for multiplicity.

**Table 3:** Secondary outcomes in ACDF and PCF patients.

Variable	ACDF (*N* = 7331)	PCF (*N* = 2038)	Difference Unadjusted (95% CI)	*p* Value ^a^
Pulmonary embolism	16 (0.2)	3 (0.1)	−0.1% (−0.3% to 0.1%)	0.753
Cerebrovascular accident	5 (0.1)	-	−0.1% (−0.1% to −0.0%)	0.999
Cardiac arrest	16 (0.2)	9 (0.4)	0.2% (−0.1% to 0.5%)	0.135
Myocardial infarction	7 (0.1)	2 (0.1)	0.0% (−0.2% to 0.2%)	0.968
Deep vein thrombosis/thrombophlebitis	30 (0.4)	3 (0.1)	−0.3% (−0.5% to −0.0%)	0.135
Systemic sepsis	22 (0.3)	23 (1.1)	0.8% (0.4% to 1.3%)	<0.001 *
On ventilator > 48 h	41 (0.6)	8 (0.4)	−0.2% (−0.5% to 0.2%)	0.422
Acute renal failure	2 (0.0)	1 (0.0)	0.0% (−0.1% to 0.1%)	0.481
Any surgical site infection	39 (0.5)	68 (3.3)	2.8% (2.0% to 3.6%)	<0.001 *
Superficial incisional	18 (0.2)	43 (2.1)	1.9% (1.2% to 2.5%)	<0.001 *
Deep incisional	9 (0.1)	18 (0.9)	0.8% (0.3% to 1.2%)	<0.001 *
Organ/space	13 (0.2)	9 (0.4)	0.3% (−0.0% to 0.6%)	0.094

Data is from the weighted analysis. Data are mean ± standard deviation or number of patients (%). The differences are mean difference and difference in proportions, along with their 95% confidence intervals. CI: confidence intervals. ^a^ Adjusted *p* values: Benjamini–Hochberg correction with a false discovery rate (FDR) of 0.05 was applied to adjust for multiplicity. The widths of the confidence intervals were not adjusted for multiple testing and may not be used in place of hypothesis testing. * Significant *p* values after adjusting for multiplicity.

**Table 4:** Differences in baseline characteristics between anterior cervical discectomy and fusion and posterior cervical foraminotomy patients stratified by 30-day re-admission and 30-day re-operation.

Characteristics	Re-Admission				Re-Operation			
	ACDF (*N* = 175)	PCF (*N* = 64)	Difference Unadjusted (95% CI)	*p* Value	ACDF (*N* = 125)	PCF (*N* = 39)	Difference Unadjusted (95% CI)	*p* Value
Age (years)	60.2 ± 11.1	56.7 ± 14.3	−3.6 (−7.4 to 0.3)	0.05	62.8 ± 10.8	56.6 ± 13.4	−6.2 (−10.8 to −1.6)	0.003 *
Female sex	76 (43.4)	34 (53.1)	9.7% (−4.5% to 23.9%)	0.166	48 (38.4)	22 (56.4)	18.0% (0.2% to 35.8%)	0.046 *
Male sex	99 (56.6)	30 (46.9)	−9.7% (−23.9% to 4.5%)	0.166	77 (61.6)	17 (43.6)	−18.0% (−35.8% to −0.2%)	0.046 *
Not specified	-	-	0.0% (0.0% to 0.0%)	0.999	-	-	0.0% (0.0% to 0.0%)	0.999
Race								
White	141 (80.6)	39 (60.9)	−19.6% (−32.9% to −6.4%)	0.001	90 (72.0)	20 (51.3)	−20.7% (−38.3% to −3.1%)	0.018 *
Black	20 (11.4)	15 (23.4)	12.0% (−0.6% to 23.4%)	0.032	19 (15.2)	15 (38.5)	23.3% (6.7% to 39.8%)	0.003 *
Asian	1 (0.6)	-	−0.6% (−1.7% to 0.5%)	0.999	2 (1.6)	-	−1.6% (−3.8% to 0.6%)	0.999
American Indian/Alaska Native	1 (0.6)	1 (1.6)	1.0% (−2.2% to 4.2%)	0.439	-	-	0.0% (0.0% to 0.0%)	0.999
Native Hawaiian/Pacific Islander	--	-	0.0% (0.0% to 0.0%)	0.999	-	-	0.0% (0.0% to 0.0%)	0.999
Other	-	-	0.0% (0.0% to 0.0%)	0.999	-	-	0.0% (0.0% to 0.0%)	0.999
Hispanic ethnicity	12 (6.9)	2 (3.1)	−3.7% (−9.4% to 1.9%)	0.207	10 (8.0)	2 (5.1	−2.9% (−11.3% to 5.5%)	0.390
Diabetes status								
Diabetes insulin	18 (10.3)	8 (12.5)	2.2% (−7.0% to 11.5%)	0.655	10 (8.0)	8 (20.5)	12.5% (−1.1% to 26.1%)	0.032 *
Diabetes non-insulin	24 (13.7)	11 (17.2)	3.5% (−7.1% to 14.0%)	0.447	15 (12.0)	7 (17.9)	5.9% (−7.4% to 19.3%)	0.312
No diabetes	133 (76.0)	45 (70.3)	−5.7% (−18.5% to 7.1%)	0.350	101 (80.8)	24 (61.5)	−19.3% (−36.1% to −2.5%	0.018 *
BMI (kg/m^2^)	30.4 ± 6.9	32.5 ± 6.8	2.1 (0.1 to 4.0)	0.042	29.6 ± 7.0	33.0 ± 6.8	3.5 (1.0 to 5.9)	0.010 *
Smoker	48 (27.4)	27 (42.2)	14.8% (1.0% to 28.5%)	0.034	32 (25.6)	12 (30.8)	5.2% (−11.2% to 21.6%)	0.428
Prior medical history								
CHF	-	-	0.0% (0.0% to 0.0%)	0.999	1 (0.8)	-	−0.8% (−2.4% to 0.8%)	0.999
Disseminated cancer	2 (1.1)	-	−1.1% (−2.7% to 0.4%)	0.999	1 (0.8)	-	−0.8% (−2.4% to 0.8%)	0.999
Steroid use	10 (5.7)	3 (4.7)	−1.0% (−7.2% to 5.2%)	0.667	8 (6.4)	-	−6.4% (−10.7% to −2.1%)	0.999
Bleeding disorder	3 (1.7)	1 (1.6)	−0.2% (−3.7% to 3.4%)	0.782	7 (5.6)	-	−5.6% (−9.6% to −1.6%)	0.999
COPD	23 (13.1)	3 (4.7)	−8.5% (−15.6% to −1.3%)	0.095	10 (8.0)	-	−8.0% (−12.7% to −3.3%)	0.999
Dialysis	2 (1.1)	-	−1.1% (−2.7% to 0.4%)	0.999	2 (1.6)	-	−1.6% (−3.8% to 0.6%)	0.999
Weight loss	-	-	0.0% (0.0% to 0.0%)	0.999	-	-	0.0% (0.0% to 0.0%)	0.999
Renal failure	-	1 (1.6)	1.6% (−1.5% to 4.6%)	0.999	1 (0.8)	-	−0.8% (−2.4% to 0.8%)	0.999
Dyspnea	13 (7.4)	2 (3.1)	−4.3% (−10.1% to 1.5%)	0.268	6 (4.8)	-	−4.8% (−8.5% to −1.1%)	0.999
Transfer from non-home	9 (5.1)	3 (4.7)	−0.5% (−6.6% to 5.7%)	0.660	12 (9.6)	3 (7.7)	−1.9% (−11.8% to 7.9%)	0.558
Functional status								
Independent	163 (93.1)	57 (89.1)	−4.1% (−12.6% to 4.4%)	0.336	114 (91.2)	32 (82.1)	−9.1% (−22.2% to 3.9%)	0.129
Partially dependent	9 (5.1)	7 (10.9)	5.8% (−2.5% to 14.1%)	0.122	8 (6.4)	7 (17.9)	11.5% (−1.3% to 24.4%)	0.037 *
Dependent	3 (1.7)	-	−1.7% (−3.6% to 0.2%)	0.999	3 (2.4)	-	−2.4% (−5.1% to 0.3%)	0.999
ASA class								
1	-	2 (3.1)	3.1% (−1.1% to 7.4%)	0.999	2 (1.6)	2 (5.1)	3.5% (−3.8% to 10.8%)	0.352
2	58 (33.1)	22 (34.4)	1.2% (−12.3% to 14.8%)	0.904	33 (26.4)	15 (38.5)	12.1% (−5.1% to 29.2%)	0.196
3	105 (60.0)	38 (59.4)	−0.6% (−14.6% to 13.4%)	0.885	71 (56.8)	23 (59.0)	2.2% (−15.6% to 19.9%)	0.860
4	12 (6.9)	3 (4.7)	−2.2% (−8.5% to 4.2%)	0.622	18 (14.4)	-	−14.4% (−20.5% to −8.3%)	0.999
RAI-rev score	20.2 ± 6.3	18.4 ± 7.1	−1.8 (−3.8 to 0.1)	0.052	21.6 ± 6.3	18.6 ± 6.9	−3.0 (−5.5 to −0.6)	0.009 *
Pre-operative lab values								
WBC (1000/mL)	7.8 ± 2.7	8.2 ± 3.0	0.4 (−0.4 to 1.3)	0.284	8.2 ± 3.5	7.82 ± 1.2	−0.4 (−1.1 to 0.4)	0.534
HCT (%)	40.6 ± 5.6	41.3 ± 4.0	0.7 (−0.6 to 2.0)	0.349	40.5 ± 5.8	42.4 ± 4.1	1.9 (0.3 to 3.5)	0.064
Platelets (1000/mL)	264.0 ± 88.7	250.5 ± 83.7	−13.5 (−37.8 to 10.8)	0.287	249.0 ± 75.6	234.4 ± 67.8	−14.6 (−39.7 to 10.5)	0.279
PTT (s)	29.7 ± 3.9	28.9 ± 4.4	−0.8 (−2.0 to 0.4)	0.182	29.4 ± 3.5	28.6 ± 2.7	−0.8 (−1.8 to 0.3)	0.212
INR	1.0 ± 0.2	1.03 ± 0.1	0.0 (−0.0 to 0.0)	0.513	1.0 ± 0.2	1.0 ± 0.	−0.0 (−0.0 to 0.0)	0.872
BUN (mg/dL)	17.4 ± 9.2	17.4 ± 11.7	−0.1 (−3.2 to 3.1)	0.958	17.5 ± 10.3	15.3 ± 6.7	−2.2 (−4.9 to 0.6)	0.196
Creatinine (mg/dL)	1.0 ± 0.7	1.0 ± 0.8	−0.0 (−0.3 to 0.2)	0.770	1.0 ± 0.8	0.8 ± 0.2	−0.2 (−0.4 to −0.1)	0.057
Albumin (g/dL)	4.0 ± 0.5	4.0 ± 0.3	0.1 (−0.1 to 0.2)	0.446	3.9 ± 0.5	4.1 ± 0.39	0.1 (0.0 to 0.3)	0.122
Specialty								
Neurosurgery	127 (72.6)	52 (81.2)	8.7% (−2.9% to 20.3%)	0.162	87 (69.6)	27 (69.2)	−0.4% (−17.0% to 16.2%)	0.892
Orthopedic surgery	48 (27.4)	12 (18.8)	−8.7% (−20.3% to 2.9%)	0.162	38 (30.4)	12 (30.8)	0.4% (−16.2% to 17.0%)	0.892

Data are mean ± standard deviation or number of patients (%). ASA: American society of anesthesiologists’ physical status; BMI: body-mass-index; BUN: blood urea nitrogen; CHF: congestive heart failure; COPD: chronic obstructive pulmonary disease; HCT: hematocrit; INR: international normalized ratio; PTT: partial thromboplastin time; WBC: white blood cells. * *p* values were not adjusted for multiplicity.

**Table 5:** Differences in outcomes between anterior cervical discectomy and fusion and posterior cervical foraminotomy patients stratified by 30-day re-admission and 30-day re-operation.

Characteristics	Re-Admission				Re-Operation			
	ACDF (*N* = 175)	PCF (*N* = 64)	Difference Unadjusted (95% CI)	*p* Value	ACDF (*N* = 125)	PCF (*N* = 39)	Difference Unadjusted (95% CI)	*p* Value
Operative time	142.0 ± 80.7	89.3 ± 43.3	−52.7 (−68.7 to −36.8)	<0.001	155.00 ± 93.28	90.8 ± 52.0	−64.2 (−87.3 to −41.1)	<0.001 *
Length of stay	3.5 ± 4.8	2.56 ± 2.59	−0.9 (−1.9 to 0.1)	0.134	9.12 ± 11.3	3.7 ± 4.5	−5.4 (−7.8 to −3.0)	0.001 *
Post-operative complications								
Pulmonary embolism	6 (3.4)	1 (1.6)	−1.9% (−5.9% to 2.2%)	0.414	3 (2.4)	-	−2.4% (−5.1% to 0.3%)	0.999
Cerebrovascular accident	2 (1.1)	-	−1.1% (−2.7% to 0.4%)	0.999	1 (0.8)	-	−0.8% (−2.4% to 0.8%)	0.999
Cardiac arrest	1 (0.6)	1 (1.6)	1.0% (−2.2% to 4.2%)	0.284	1 (0.8)	-	−0.8% (−2.4% to 0.8%)	0.999
Myocardial infarction	-	1 (1.6)	(−1.5% to 4.6%)	0.999	3 (2.4)	-	−2.4% (−5.1% to 0.3%)	0.999
Deep vein thrombosis/thrombophlebitis	10 (5.7)	2 (3.1)	−2.6% (−8.1% to 2.9%)	0.318	8 (6.4)	-	−6.4% (−10.7% to −2.1%)	0.999
Systemic sepsis	4 (2.3)	17 (26.6)	24.3% (13.3% to 35.3%)	<0.001	6 (4.8)	16 (41.0)	36.2% (20.3% to 52.2%)	<0.001 *
On ventilator > 48 h	4 (2.3)	1 (1.6)	−0.7% (−4.5% to 3.0%)	0.986	18 (14.4)	-	−14.4% (−20.5% to −8.3%)	0.999
Acute renal failure	-	1 (1.6)	1.6% (−1.5% to 4.6%)	0.999	2 (1.6)	-	−1.6% (−3.8% to 0.6%)	0.999
Any SSI	18 (10.3)	28 (43.8)	33.5% (20.5% to 46.4%)	<0.001	15 (12.0)	25 (64.1)	52.1% (36.0% to 68.2%)	<0.001 *
Superficial incisional	4 (2.3)	5 (7.8)	5.5% (−1.4% to 12.4%)	0.076	4 (3.2)	5 (12.8)	9.6% (−1.3% to 20.6%)	0.029 *
Deep incisional	6 (3.4)	18 (28.1)	24.7% (13.4% to 36.0%)	<0.001	6 (4.8)	15 (38.5)	33.7% (17.9% to 49.4%	<0.001 *
Organ/space	9 (5.1)	6 (9.4)	4.2% (−3.6% to 12.1%)	0.180	6 (4.8)	7 (17.9)	13.1% (0.5% to 25.8%	0.016 *
Any re-admission	-	-	-	-	52 (41.6)	34 (87.2)	45.6% (32.0% to 59.2%)	<0.001 *
Re-admission associated with SSI	12 (6.9)	27 (42.2)	35.3% (22.7% to 48.0%)	<0.001	6 (4.8)	22 (56.4)	51.6% (35.6% to 67.7%	<0.001 *
Superficial incisional	3 (1.7)	3 (4.7)	3.0% (−2.5% to 8.5%)	0.181	1 (0.8)	2 (5.1)	4.3% (−2.8% to 11.4%)	0.087
Deep incisional	6 (3.4)	17 (26.6)	23.1% (12.0% to 34.3%)	<0.001	5 (4.0)	14 (35.9)	31.9% (16.4% to 47.4%)	<0.001 *
Organ/space	3 (1.7)	6 (9.4)	7.7% (0.3% to 15.0%)	0.014	-	6 (15.4)	15.4% (4.0% to 26.7%)	0.998
Re-operation	52 (29.7)	34 (53.1)	23.4% (9.5% to 37.4%)	0.001	-	-	-	-

Data are mean ± standard deviation or number of patients (%). CI: confidence interval; SSI: surgical site infection. * *p* values were not adjusted for multiplicity.

## Data Availability

All data is available in the ACS-NSQIP database. Please reach out to the corresponding author J.G. with any inquiries.
